# Performance-based financing in three humanitarian settings: principles and pragmatism

**DOI:** 10.1186/s13031-018-0166-9

**Published:** 2018-06-27

**Authors:** Maria Paola Bertone, Eelco Jacobs, Jurrien Toonen, Ngozi Akwataghibe, Sophie Witter

**Affiliations:** 1 ReBUILD & Institute for Global Health and Development, Queen Margaret University, Edinburgh, UK; 2 Royal Tropical Institute (KIT), Amsterdam, the Netherlands

**Keywords:** Performance based financing, Implementation process, Fragile and conflict-affected settings, DR Congo, Central African Republic, Nigeria

## Abstract

**Background:**

Performance based financing (PBF) has been increasingly implemented across low and middle-income countries, including in fragile and humanitarian settings, which present specific features likely to require adaptation and to influence implementation of any health financing programme. However, the literature has been surprisingly thin in the discussion of how PBF has been adapted to different contexts, and in turn how different contexts may influence PBF. With case studies from three humanitarian settings (northern Nigeria, Central African Republic and South Kivu in the Democratic Republic of Congo), we examine why and how PBF has emerged and has been adapted to those unsettled and dynamic contexts, what the opportunities and challenges have been, and what lessons can be drawn.

**Methods:**

Our comparative case study is based on data collected from a document review, 35 key informant interviews and 16 focus group discussions with stakeholders at national and subnational level in the three settings. Data were analysed in order to describe and compare each setting in terms of underlying fragility features and their implications for the health system, and to look at how PBF has been adopted, implemented and iteratively adapted to respond to acute crisis, deal with other humanitarian actors and involve local communities.

**Results:**

Our analysis reveals that the challenging environments required a high degree of PBF adaptation and innovation, at times contravening the so-called ‘PBF principles’ that have become codified. We develop an analytical framework to highlight the key nodes where adaptations happen, the contextual drivers of adaptation, and the organisational elements that facilitate adaptation and may sustain PBF programmes.

**Conclusions:**

Our study points to the importance of pragmatic adaptation in PBF design and implementation to reflect the contextual specificities, and identifies elements (such as, organisational flexibility, local staff and knowledge, and embedded long-term partners) that could facilitate adaptations and innovations. These findings and framework are useful to spark a reflection among PBF donors and implementers on the relevance of incorporating, reinforcing and building on those elements when designing and implementing PBF programmes.

**Electronic supplementary material:**

The online version of this article (10.1186/s13031-018-0166-9) contains supplementary material, which is available to authorized users.

## Background

Performance based financing (PBF) schemes aim to improve health service delivery by providing bonuses to service providers (usually facilities, but often with a portion paid to individual staff) based on verified quantity of outputs produced, modified by quality indicators [1]. Such programmes have been increasingly implemented across low and middle income countries in the past decade with considerable external financing from multilateral, bilateral and global health initiatives [2]. Although it is clear from the early studies that PBF is unlikely to be a homogenous intervention and that its modalities and effects will be dependent on context [3], the literature on PBF has been surprisingly thin in its discussion of how different contexts may influence PBF programmes [4]. In an effort to address this gap, a recent hypothesis-led review, focussing on fragile and conflict-affected states (FCAS) where PBF programmes have particularly proliferated, gathered the existing evidence available in grey and published literature on how the FCAS context may influence the adoption, adaption, implementation and health system effects of PBF [5]. The review found that evidence on the interaction between PBF and context is still limited and pointed to some critical issues that deserve further attention. In particular, it highlighted that, contrary to expectation, PBF design was relatively homogenous across FCAS settings, with the notable exception that in humanitarian settings some adaptations were emerging. As these adaptations are only partially described in the grey literature and not analysed in published studies, they were deemed to merit in-depth exploration, which is the objective of this paper.

The questions we address are of high policy relevance as PBF continues to be used as a dominant financing modality by donors, such as the World Bank [6] and remains controversial [7]. Reflecting on how and why PBF can be adapted to context (both at design and implementation stage), and on which are the factors enabling such adaptations, is extremely relevant for the operational practice of PBF in all contexts. This is even truer for FCAS, whose challenging environment poses specific questions for adaptation and innovation. An estimated 125 million people worldwide are in need of humanitarian support [8] with a growing number of conflicts, many of which remain unresolved for years, leading to long-term vulnerability for the populations in these areas. At global level, there is increasing interest in effective financing mechanisms to support access to basic services for conflict-affected populations [9], and this article contributes to these academic and policy debates.

In this paper, using case studies from three humanitarian settings – northern Nigeria, the Central African Republic (CAR) and South Kivu in the Democratic Republic of Congo (DRC) - we examine why and how PBF has been adapted to those unsettled and dynamic contexts, what the opportunities and challenges have been, and what lessons can be drawn. In the absence of sufficient data, we do not address the issue of whether PBF is effective in humanitarian contexts; our study aims to contribute to the literature on PBF design and implementation, rather than on its impact.

## Methods

This research adopted a comparative case study design, where the case studies allow exploring a phenomenon in context (especially when the context is an integral part of what is being studied) and the comparison strengthens explanatory power and analytic generalisability [10–12]. Data were collected through a review of documents and a series of key informant interviews (KIIs) and focus group discussions (FGDs) in the three settings. A common protocol was developed so that data would be comparable across cases. The topic guide for KIIs and FGDs, although tailored to each respondent, group and setting, overall focused on the relation between fragility and health systems, the description of PBF programme(s) and the process of their introduction and development over time, challenges in implementation, and adaptations and innovations introduced at design or implementation stage to respond to conflict and humanitarian crisis (Additional file 1).

### Data collection

The document search targeted published and unpublished documents that describe the PBF programmes, their design and implementation, provided information on effectiveness and/or challenges of the programme, and detailed the adaptations made over time. Documents were retrieved through the database put together for the literature review on PBF in fragile settings [5] to be reanalysed for the purpose of this paper, but also through key informants and direct knowledge of the context. Documents reviewed included a few published articles, but were mostly unpublished, health sector-related (e.g., policies and strategies) and PBF project documents (e.g., implementation manuals, sample of contracts, list of indicators, internal and external evaluations, presentations, annual reviews). In total, 25 documents were reviewed for South Kivu, 24 for Nigeria and 16 for CAR.

Ethics approval was obtained from Queen Margaret University’s Research Ethics Panel, and fieldwork was carried out between June and November 2017. FGDs and KIIs were carried out in person in Nigeria (JT, NA), remotely via phone, Skype or WhatsApp for DRC (MPB), and a mix of in-person KIIs and FGDs, and phone interviews for CAR (EJ). The choice between FGDs and KIIs was made based on what was best adapted to capture the elements included in the topic guide, as well as to take advantage of existing opportunities, such as meetings already organised which gathered stakeholders.

Participants were identified through the document review (e.g. authors of a report), as well as by contacting the PBF implementing agency and/or the Ministry of Health (MoH) in the relevant countries. A snowball technique was also adopted by asking interviewees to suggest others. In all settings, participants were selected purposefully, with the aim of being as comprehensive as possible, focusing on those involved in PBF design and/or implementation and, where relevant and possible, also other actors not directly involved in PBF but responsible for service organisation and delivery in areas where PBF operated. As much as possible, different levels of the health system were included in the KIIs/FGDs. In DRC, interviews focused on actors at provincial level in South Kivu, although international respondents were also included (e.g., consultants and project managers’ at headquarters). In CAR, respondents included international, national and local (district) actors and in Nigeria, central level decision-makers and members of the PBF project's implementation unit in the MoH, managers of the implementing agency at central level and staff at operational level, including MoH staff. In total, 34 KIIs and 18 FGDs were carried out. Table 1 provides a summary of the FGDs and KIIs, and an overview of the characteristics of the participants.

**Table 1:** Summary of FGDs and KIIs carried out

Country	Method	Type of interviewees / participants	Num. of KIIs / FGDs	Total
DRC	KIIs	Implementing organisations	6	KIIs = 13
		Consultants	2	
		Health administration at provincial and zonal level	3	
		Other organisations	2	
CAR	KIIs	Implementing organisations	4	KIIs = 10 FGDs = 6
		Consultants	2	
		Other organisations (international and national)	4	
	FGDs	Health administration at national and district level	2	
		Other organisations	4	
Nigeria	KIIs	Central level MoH decision-makers	3	KIIs = 12; FGDs =10
		Implementing agency managers	3	
		Operational level – MoH and implementing agency	6	
	FGDs	Central level MoH decision-makers	2	
		Implementing agency managers	4	
		Operational level – MoH and implementing agency	4	

### Data analysis

KIIs were recorded and/or detailed notes were taken during interviews and FGDs, and analysis was carried out based on those notes. Documents and notes from KIIs/FGDs were manually analysed by the author/team focusing on each setting (JT, NA for Nigeria, MPB for DRC and EJ for CAR) using thematic analysis and based on a list of predefined categories, which was developed based on an existing study [13]. Summary reports were prepared independently for each case study. During a 3-day workshop in October 2017, the research teams presented the findings for each country, which were charted in a table with columns referring to cases and rows to original categories, also adding new categories/themes which had emerged (Table 2). The table allowed comparative analysis, highlighting differences and emerging patterns across settings. The results section below is organised following the categories of Table 2.

**Table 2:** Pre-identified and emerging themes used for the comparative analysis of case studies

Context	Elements of the broader context
	Nature of the conflict and fragility features
	Pre-existing political settlements
	Effects of conflict on health system
Formulation and design of PBF	Period/duration of the PBF programme
	Implementers and funders
	PBF design and institutional arrangements
	Facilities and services covered
	Key actors and organisations driving or blocking PBF introduction
	Nature of the debate around the introduction of PBF
Implementation of PBF	Innovations/adaptations to PBF and coping strategies in acute crisis
	Coordination with other actors
	Role of communities

## Results

### Contexts

#### The underlying conflicts and features of fragility

All three settings have experienced intermittent conflict, which is either on-going or in a fragile lull period. In the East of the DRC, South Kivu, with a population of around 5 million [14], has been heavily involved in the First (1996–1997) and Second (1998–2003) Congo Wars, and subsequently experienced protracted conflicts and persisting violence, with a number of rebel forces competing for political power and the control of natural resources, including minerals and land [15]. Identity narratives, territorial claims and the influence of neighbouring countries help perpetuate the conflict [16]. In this study, we mostly focus on two among the 5 health zones where PBF has been implemented (out of a total of 34 health zones in the province) – those of Shabunda and Lulingu, which together form the administrative unit of Shabunda territory, the largest and most isolated territory of South Kivu. PBF was implemented in Shabunda and Lulingu between 2008 and 2012, when the area was moving towards (relative) peace and stability and the main humanitarian health organisations had moved out. Acute crises were only sporadic, but a few periods of instability occurred especially around 2009 with increased fighting and displacement of civilians [17].

Fragility features in South Kivu -and more broadly across the DRC- include the quasi absence of state services, such as justice, health care and security, for which local populations have to rely on a network of state and non-state actors [18]. Roads, communications, markets and financial institutions are lacking or run-down, which poses a challenge for service delivery but also for PBF.

CAR, which has a population of around 4.6 million [19], also faces a protracted crisis with acute phases, including at the moment in the North and East, where violence flared up again in early 2017. Although the recent conflict dates to the Séléka rebel coalition overthrow of the government in early 2013, instability has been raging at varying levels of intensity throughout CAR’s existence as an independent country. The root causes include a scramble over resources (diamonds, timber, gold, and land), fuelling inter-communal violence, aggravated by historic grievances and revenge [20, 21]. Economic tensions, including between settled farmers and (neo-)pastoralists, have also been reinforced by an instrumentalisation of religious and ethnic differences and resentment against the former colonial power, France, and the United Nations Multidimensional Integrated Stabilisation Mission in the Central African Republic (MINUSCA).

Fragility in CAR brings similar challenges to South Kivu and the country has been described as a ‘phantom state’ [22] or ‘caricature of a state’ [23]. The extreme deprivation and limited scale of financial services has meant that cash is reported by key informants to have almost disappeared from certain regions. Most of the economy is informal, survival-based and even criminalised in parts, which directly affects areas essential to PBF such as drugs purchases.

Both DRC and CAR have been described as neo-patrimonial, predatory states [24], in which power is focused on individuals and their protective networks which extract resources as a source of power and control [25]. Remoteness from the capital and lack of centralised control or systems creates de facto local autonomy [26] and the privatisation, or even de-institutionalisation, of the public sphere [27].

Northern Nigeria has suffered from deepening insecurity since 2009, as the result of the activities of Boko Haram. Boko Haram means ‘western education is forbidden’, because of the group’s aversion to western civilisation, including western healthcare. The insurgency has involved bombings, armed raids and robberies, rising in intensity since 2012 [28, 29]. By 2017, over 20,000 people had been killed, more than 2 million displaced, and over 6 million were in need of humanitarian assistance [30]. The area saw a collapse in security and health services, along with trade routes, markets, education and many of the determinants of health, such as water, sanitation and food security. There was massive population displacement into internally displaced persons (IDP) camps. In this study, we focus on Adamawa State (population of 4.5 million [31]), where PBF is implemented. Here, the impact of Boko Haram’s activities was compounded by political instability, with four different state governors and executive councils taking power within one year (mid-2014 to mid-2015). As in other settings, the poorer populations were worst affected by the disorder, and poverty and political marginalisation of the North within Nigeria remain as underlying risk factors.

#### Implications of conflict and fragility for the health systems

The effects of the conflict on the local health system had many shared features across the three settings, although the policy environment and responses differed (Table 3).

**Table 3:** Summary of implications of conflict and fragility for the health systems

	South Kivu / DR Congo	Central African Republic	Adamawa State / Nigeria
National governance and leadership	• Conflict exacerbated pre-existing weaknesses related to lack of governance and underfunding	• MoH lost its leadership role to donors and NGOs	• Structured federal system with effective decentralisation • Federal and state governments’ efforts to strengthen primary health care delivery
Consequences of conflict on service delivery	• Violent episodes have left infrastructure destroyed, equipment pillaged and led to lack of staff in some areas	• By 2016 27% of health facilities were partially or fully destroyed, and of all functioning facilities only 22% had a source of energy and 43% running water	• Insurgency left only 37% of facilities functional with limited staff, a break-down in governance and facing disease outbreaks
Healthcare financing	• No fee exemption policies (except for some vertically-funded preventative services) • Reliance on user fees and external interventions	• Since 2014, externally funded free healthcare policy for women (covering perinatal services), children and ‘emergency’ services	• User fees in place generally, though lifted at the height of the crisis in 2014

In South Kivu, the conflict had exacerbated pre-existing health system’s weaknesses related to fragility and underfunding, with low levels of staff training, drug stock-outs and bad quality of drugs, poor governance and lack of supervision of health facilities [32]. Particularly relevant is the long-standing phenomenon of the “*financement ascendant*” (also known as ‘*la pompe’ -* [33]) by which a proportion of the earnings through user fees are levied on facilities by the Zonal Health Management Team (ZHMT) in order to support their own costs. Similarly, the provincial authorities rely on the Zones for their funding.

As a consequence of the volatile and instable situation, in CAR by mid-2016 nearly half the population were in need of humanitarian assistance and infrastructure was severely damaged (see Table 3) [34, 35]. At central level, the MoH was seen by key informants to have lost its leadership role, with donors and NGOs left to pursue their own objectives in an uncoordinated way. With external funding, a policy of free healthcare for women, children and ‘emergency’ services was instituted nationwide at the height of the crisis, though later scaled down in stable areas but still in place in the most insecure areas [36]. Additionally, key informants reported that the Central Medical Store was not functioning due to mismanagement and corruption and its future was uncertain, with stakeholders holding competing visions for it.

In contrast with the other settings, Nigeria had a more structured federal system with effective decentralisation to state level for functions such as healthcare. Nevertheless, Adamawa State was already less developed in its health system compared to other regions prior to the insurgency, and the conflict created huge damage [37, 38]. Despite the conflict, however, the central and state-level administrations remain relatively functional and have been attempting to strengthen the health system. In particular, the management and delivery of primary health care (PHC) was reformed nationwide in 2011 to reduce fragmentation following the “Primary Health Care Under One Roof” policy, which established the creation of a single State PHC Development Agency (SPHCDA), to provide coordinated leadership [39].

### Formulation and adoption of PBF programmes

Against these contexts, all characterised by extreme fragility with phases of acute instability, the PBF programmes were designed and adopted. We describe these processes here.

In South Kivu, discussions about the introduction of PBF started around 2005–2006 and were led by the Dutch NGO Cordaid (Caritas Netherlands) (and their consultant), which had been implementing one of the first PBF programmes in neighbouring Cyangugu province, Rwanda. Initially, PBF covered 2 health zones (Katana and Idjiwi – [40]) and was later expanded to others (including Shabunda and Lulingu in 2008–2012), covering a maximum of 5 health zones and a population of 750,000 [41, 42]. As of mid-2017, Cordaid’s PBF project has been discontinued, although PBF continues to be implemented in South Kivu under other projects (most notably, with World Bank’s funding). Cordaid’s PBF project in South Kivu was funded largely by the Dutch Embassy, but also from Cordaid’s own funds and other donors, providing about 2–3 USD per capita [17, 40]. Initially, Cordaid worked in collaboration with the Diocesan Medical Bureau (*Bureau Diocésain d’Œuvres Médicales*, BDOM), though this was later changed as the BDOM was perceived to have a conflict of interest since it is one of the major health providers in the province. A new purchasing agency (*Agence d’Achat des Performances*, AAP) was created to implement the project, with the status of national NGO, staffed by Congolese personnel and funded by Cordaid^1^. The creation of a local AAP is considered a ‘mixed arrangement’ and, at the time of its conception, constituted an original feature of the PBF project in South Kivu [43], in contrast to the majority of the early PBF projects where the role was played by the implementing NGO or by a unit attached to the MoH. The combination of multiple roles under the responsibility of the AAP was seen by some key informants as against the PBF ‘principle’ of separation of functions (by which it is understood “a clear demarcation between purchasing, fund-holding, [service] provision, regulation and community voice” with the aim of reducing conflicts of interest and increasing transparency and accountability ([1]: p.43)). Key informants considered that it emerged and was acceptable only in light of the difficult context in which the project operated. Details of the PBF design and institutional arrangements in the three settings are provided in Table 4. It is important to note that facilities had a strong autonomy on most decisions concerning their management, including on the use of PBF funds, sharing of the performance-based payments and other incomes, the procurement of drugs and equipment as well as hiring and firing of some staff (those paid with facility revenues). The autonomy seems largely de facto and derived from the history of state disengagement in funding and managing the health sector [41].

**Table 4:** Design features of the PBF programmes across the three settings

	South Kivu / DR Congo	Central African Republic	Adamawa State / Nigeria
Funder(s)	Dutch Embassy, Cordaid, other donors (varying over time)	Current PBF programmes: • European Union / Fonds Bekou • World Bank (PASS)	World Bank
Period of implementation	2005–2017 (with varying geographical coverage)	• 2015-ongoing • 2016-ongoing	End of 2011 - ongoing
Who is included/ incentivised?	– Facilities (primary and secondary; public, private and faith-based) – Zonal Health Management Teams (ZHMTs) – Provincial authorities (later on)	– Facilities (primary and secondary; public, private and faith-based) – District Health Management Teams (DHMTs) – Regional and national health authorities – [CHWs sub-contracted by health facilities for outreach activities]	– Facilities (primary and secondary; public and faith-based)
Indicators and services included (facility level)	Indicators and bonus attached varied over time depending on budget available and focus of donor(s). Overall, within the national basic package of services forprimary and secondary levels.	The service package is harmonised across PASS and Fonds Bekou programmes, and based on the national basic package	Basic package, including vaccination, assisted deliveries, consultations for under-5s, quality of care
Institutional arrangements:	Agency responsible:		
- contracting	*Agence d’Achat des Performances* (AAP) for facilities and ZHMTs (Cordaid for provincial level)	• Fonds Bekou: Cordaid • PASS: international implementing agencies (AEDES and Cordaid) for facilities + MoH’s Project Implementation Unit (PIU) for health regulation	Project Implementation Unit (PIU) within the State’s PHC Development Agency (SPHCDA), with international technical assistance from Oxford Policy Management (OPM) for the first two years before it started operating autonomously
- quantitative verification	AAP	• Fonds Bekou: Cordaid • PASS: international implementing agencies (AEDES and Cordaid)	SPHCDA (initially with support from international TA)
- qualitative verification	ZHMTs/Provincial teams	DHMTs	SPHCDA (initially with support from international TA)
- community verification	Community Based Organisations, contracted by AAP	Community Based Organisations, contracted by implementing agencies	Grassroot NGOs/CSOs, contracted by the SPHCDA
- fund-holding and payment	AAP	• Fonds Bekou: Cordaid • PASS: PIU with the exception of 10% of facilities which do not have bank accounts and therefore are paid via the implementing agencies.	SPHCDA
Fee exemptions for vulnerable populations	Initially not planned, but were later introduced [42]. Project’s evaluations noted that they were largely not functioning [41, 47]	• Yes for PASS project only (KII; [44]).	Introduced in Adamawa State only
Equity bonus across areas	No	• PASS: indigents are exempted from fee-paying, for which health facilities are compensated. Identification of indigents is done at community level without standardised criteria.	No

In the documents reviewed, there is no evidence of a debate on the suitability of PBF in South Kivu by local stakeholders before its introduction, which may be explained by the weakness of local institutions and also the way in which NGOs tend to operate in conflict-affected and humanitarian settings, where they compete for donors’ funding and for influence on health authorities and tend to operate in parallel, not engaging each other in debate about the relevance of their approach or programme [43]. However, documents and KIIs contain several *post-hoc* justifications of why PBF was suitable in the context of South Kivu. Explanations relate to the absence of state funding [43], as well as to the perceived pre-existing entrepreneurial ‘franchise’ fashion of service delivery given the state’s absence [41] and to the de facto autonomy of providers, which allows PBF to be designed and to operate more freely (KII).

In CAR, PBF has been implemented since 2009 through a series of pilots, also led by Cordaid. At the time of this study, two main projects were ongoing. The first is supported by the European Union (Fonds Bekou pooled funding) since 2015 and implemented by Cordaid, covering about 341,600 people, while the second is the World Bank-funded *Projet d’Appui au Système de la Santé* (PASS) which started in 2016. Under the leadership of the Ministry of Health’s Project Implementation Unit (PIU), two international agencies are responsible for PASS implementation – Cordaid (which covers a population of 1.2 million) and AEDES (0.5 million). PASS has a budget of 5 USD per capita, compared to € 11 per capita for the EU/Fonds Bekou-funded PBF (KII; [44]).

In terms of PBF adoption process, it is evident from our interviews that the early PBF pilots and the lead consultant (the same person as in South Kivu) played a key role in influencing MoH staff at high level, including the Minister. However, other actors retained divergent perspectives. While the World Bank is seen by key informants as extremely supportive of PBF (also given its role in financing and supporting PBF in other countries – [5]), the European Union remained neutral (indeed, the Fonds Bekou funds multiple projects in the health sector, of which only one is a PBF scheme) and other institutions -such as the World Health Organisation- were viewed as largely unsupportive. However, there was limited debate before PBF introduction and the programmes went ahead. After its introduction, there was some opposition in particular from humanitarian NGOs, which saw PBF as unsuitable for a FCAS. Opposition to PBF was also voiced by those who oppose charging user fees for service delivery (which in CAR was seen as incompatible with PBF by most stakeholders on both sides of the argument) and those in favour of the re-establishment of the Central Medical Store (which was also seen as incompatible with PBF by many).

In Nigeria, PBF was introduced as a pilot at the end of 2012 with funding from the World Bank and is due to continue until mid-2018. The pilot covers three States (Adamawa, Nasarawa and Ondo) with a combined population of 11.6 million and a per capita budget of 14 USD [45]. In Nigeria, the process of PBF introduction was somewhat different, though still initially dominated by external players. The World Bank (through consultants) presented the approach in December 2011, and a study tour to Rwanda for Nigerian government stakeholders was funded. The MoH bought into the idea as a viable option to strengthen its new PHC policy. However, it recognised that the model had to be substantially adapted to the specificities of the Nigerian context, and in particular its decentralisation (Table 4 presents further information on the PBF programme design).

### Implementation of PBF

#### Innovations and adaptations of PBF, and strategies to cope with acute crisis

Our analysis revealed a number of adaptations which were made to the PBF projects in order to address the challenges due to the complex contexts in which they operated, in particular during violent periods. They are described in Table 5 below.

**Table 5:** PBF innovations and adaptations during crisis

	South Kivu / DR Congo	Central African Republic	Adamawa State / Nigeria
Coping with acute crisis	(*Shabunda and Lulingu health zones in 2009*): “Stay put” (rather than evacuate personnel) to build local trust and relations with the authorities in health and other sectors, but also with rebel forces when needed. The AAP was composed of local staff with established knowledge in the area, which may have helped with this strategy (KIIs)	Negotiations with all sides, including armed groups (tactics included offering free care to armed groups although this became more challenging as violence intensified).	• Few managers continued to provide health services to the non-displaced populations in conflict-affected areas, and later claimed PBF subsides • Creation of 5 PBF-funded mobile clinics to provide services in conflict-affected areas, with ‘hit and run’ approach – moving to key spots when the situation allowed to deliver first-line care and transferring critical cases to facilities in safer areas. • Armed hunters trained to carry out community health worker functions for those who had remained in the villages.
Procurement	Direct procurement of drugs and equipment for facilities, given the absence of functioning markets	(Fonds Bekou) Direct procurement of drugs and materials for facilities via a faith-based supplier, given the absence of functioning markets or Central Medical Store (this happened despite the stark debate going on in Bangui in which PBF was seen as incompatible with ‘push’ procurement systems) (KIIs)	Drugs purchased and imported from neighbouring Cameroon. PBF funding used to pre-finance drugs and essential supplies, later reimbursed with non-performance based cash transfers by other donors
Staff recruitment	–	(Fonds Bekou) Cordaid directly helped facilities to recruit qualified staff, given shortages and the underdeveloped labour market	Nationwide, the State agency for PHC recruited specific PBF staff. In Adamawa State, additional health staff was recruited for the mobile clinics
Funding rehabilitation and construction	• Flexible provision of non-performance based, advance funding (*bonus de demarrage*), not paid in cash but used by AAP to purchase construction materials not available locally • Mobilising communities’ labour and locally-available materials (sand, stones, bricks) ([41]; KIIs).	Under both Fonds Bekou and PASS programmes: direct support for rehabilitation and construction. • Fonds Bekou: more space for non-performance based funding • PASS: requests can be made to a ‘quality improvement fund’. However, several key informants perceived these measures to be insufficient since, given the badly functioning markets and the low number and skills of staff, funds are often underutilised and inputs, rather than cash, was seen as more effective in such context (KIIs; FGDs).	WB-funded PBF programme and other (non-PBF) programmes funded rehabilitation and construction, once Boko Haram had left the area.
PBF payment	Cash to facilities in absence of banking infrastructure. AAP staff distributed PBF payments to facility staff during zonal meetings or carried cash to facilities, at high personal risk (KIIs).	Cash to facilities in absence of banking infrastructure	Cash payments when no banking facility is available
PBF verification	Payments without verification (KIIs)	Payments were made at times without verification	Payments at times made without verification (FDG)
Dealing with internally displaced populations (IDPs)	Free care provided to about 20,000 IDPs. Free care was subsidised by increasing by 10–40% the PBF bonus for facilities most affected ([61]; KIIs)	Free care to IDPs in emergency areas.	Nearby facilities used PBF funds to sub-contract newly set-up clinics operating in IDP camps. Teams of 4–5 health workers living in the IDP camps or purposefully transferred from the SPHCDA were subcontracted to staff these outreach clinics, where care was provided for free to registered IDPs. Thanks to the PBF programme, a system to register IDPs was developed.

Additionally in South Kivu, we noted that (beyond the adaptations related to the acute crisis in Shabunda and Lulingu health zones described in Table 5), other innovations emerged more broadly, linked to the general fragility and ‘statelessness’. As the project evolved, extensive contracting was developed for the regulatory authorities at zonal and provincial level directly with the AAP or Cordaid, rather than between levels of the health hierarchy. This was seen by many key informants as a way to fund these agencies (which receive little or no public funding) and eliminate the need for *financement ascendant* [43], but - it appears - also to deal with the absence of the state, by bypassing and substituting central-level authorities and gaining more direct control of the activities at provincial and zonal levels. PBF was also later expanded to other sectors, including education, roads/infrastructure, and justice and security [41, 46] by contracting service providers (schools and teachers, communities engaged in construction and rehabilitation, courts and police), but also the relevant administrative authorities up to the provincial governorate. Initially, the rationale was that these sectors were considered as bottlenecks for health service delivery, but later on the conceptualisation broadened to include an explicit ‘state-building’ function beyond service delivery, under the “*Approche PBF pour le renforcement de l’Etat*” (PBF for state-building) project, which covered all those sectors [47]. Interestingly, the AAP states its mission is “to promote the social contract between the state and communities in ensuring access of citizens to quality social services, through the promotion of the PBF approach” ([48]:p.5). While some examples exist in other settings of PBF in education and water and sanitation sectors, to our knowledge such broad extension of PBF, both in operational and in conceptual terms, was unique to South Kivu. The experience was relatively short lived, pursued somewhat ‘intuitively’ and pragmatically by Cordaid [47], and little work exists to evaluate whether it represented a successful attempt to reinforce the state (though admittedly only focused on local level institutions) or is not sustainable in the long term (KII).

Underlying all the innovations (both those listed in Table 5 and those described above) in South Kivu was the small-scale and NGO-led nature of the projects which allowed for flexibility during design and implementation. Key informants recognised that such flexibility was critical to the survival (and, in their views, the success) of the project, given the dynamic and fragile setting. In CAR, more flexibility was allowed in the Fonds Bekou programme, whereas the PASS project had a more rigid operating manual. Despite that, as Table 5 shows, even under PASS, implementers had to introduce some degree of adaptation to cope with the complex environment. In Nigeria, the approach of the PBF programme since inception had been that of ‘learning by doing’, where (in contrast with the cases of CAR and South Kivu) state-level (SPHCDA and MoH) staff took a substantive lead with some support from international technical assistance. Boko Haram’s insurgency meant that further adjustments were needed in Adamawa State to cope with insecurity and shifting populations.

#### Coordination with other actors

Lack of coordination was a theme that emerged prominently in the interviews on South Kivu, particularly concerning coordination between development and humanitarian NGOs, which were seen as bypassing rules and procedures and putting in place short-term measures, such as providing free services which could be disruptive in the longer term [25]. Most interviewees told of clashes with humanitarian organisations, which happened during the 2009 crisis in Shabunda, when a humanitarian NGO returned with the intention of bringing in expatriate staff and providing free care for the entire population, even if only for a brief period, to the same area where Cordaid was supporting facilities with cash under the PBF project and another development NGO was providing drugs and commodities. After lengthy negotiations, a division of areas of influence was agreed, with the humanitarian NGO supporting three facilities and providing free services, and the development NGOs continuing their programmes in the remaining facilities, where fees were levied to the resident population but with IDPs exempted (KIIs).

Similar ideological tensions and coordination failures were reported in CAR, which in some cases resulted in duplication of support to some facilities (KII; FGD). However, there were also examples of practical cooperation on the ground, such as collaboration in the logistics of drug procurement and distribution, division of tasks in districts covered by multiple actors (e.g. Cordaid contracting health centres, where MSF is supporting the district hospital, and Cordaid providing output-based, PBF support to health facilities that the Red Cross supports with inputs) (KII; FGD).

The experience in Adamawa State presents a different case. There, the strong local leadership provided by the SPHCDA enabled tensions between different actors and approaches to be overcome and ensured multi-sectoral and coordinated responses. Monthly meetings were organised by the SPHCDA (responsible for PBF implementation), which included also donors (such as, IOM, IRC, ICRC) in charge of providing humanitarian assistance, and the (re)definition of roles and responsibilities linked to PBF structures helped practitioners set up some measure of order in a chaotic conflict environment (FGD; [49]). For example, a ‘single data registry’ was developed to register IDPs to enable PBF verification, but was also useful to other agencies to provide services and avoid duplication of care.

#### Role of communities

One of the initial hypotheses for PBF in fragile settings was that, given the lack of state structures and funding as well as the programme focus at community level (for example, through community verification and the health committees in CAR and South Kivu), PBF could be effective in reinforcing social accountability links at the local level. In fact, no evidence of this emerged in the three contexts analysed. This is possibly due to the fact that local accountability was poorly defined [47] and the expectations were perhaps too ambitious -that communities could play a variety of roles, such as verification, participation, lobbying for population rights, supervision and conflict mediation – in particular given the disruption of communities and community cohesion during acute crises.

However, the role of communities emerged in different ways, in particular in South Kivu where communities were involved by the AAP to support the rehabilitation of health facilities (as well as schools, roads and other infrastructures). A key informant stressed that the consideration that staff and communities had for ‘their’ health centre during conflict and violence was different between the PBF facilities (that they had contributed to build, rehabilitate and purchase equipment for) compared to other facilities where equipment was given as inputs by donors. In the first case, staff would hide and protect equipment, while in the latter there were instances where such equipment was lost, stolen or sold.

## Discussion

**Fig. 1:**
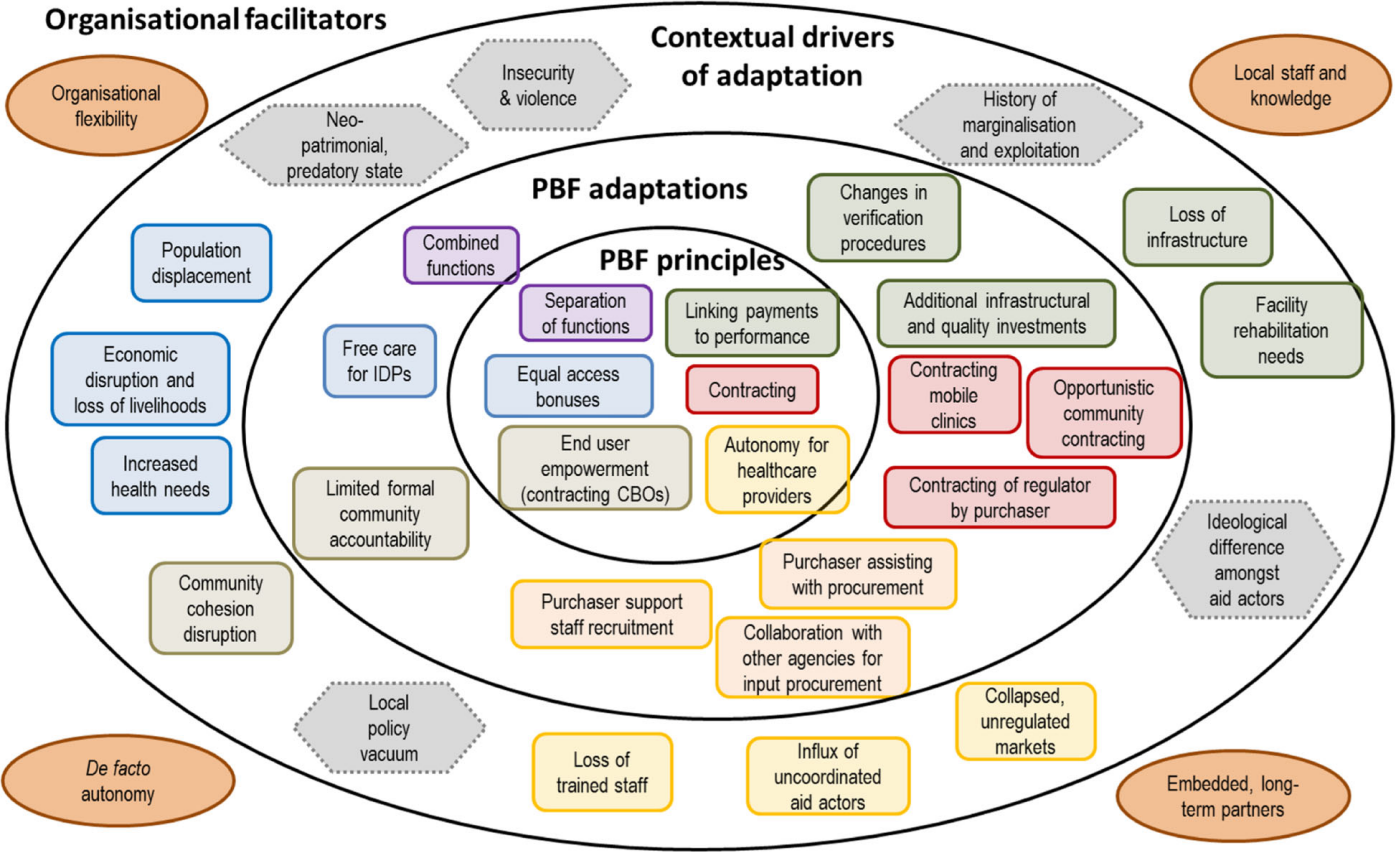
Adaptations of PBF in three humanitarian settings, their drivers and facilitators. Source: inner circle [52]; outer circles: authors, based on study findings. Examples of “PBF adaptations”, and their respective “contextual drivers”, are mapped against PBF principles by using the same colour; “contextual drivers” in grey, dotted lines are general ones. “Organisational facilitators” also refer generally to all adaptations

Conflict-affected, insecure and fast-changing contexts are not on the face of it promising environments to implement PBF. It is therefore interesting to understand how and why PBF is being adopted in such settings, and how it is adapted to them. Our study provides a first exploration of these questions in three humanitarian settings and builds on an earlier study which analysed the inter-relationship between PBF and FCAS contexts [5] . The analysis reveals interesting patterns across the cases, but also important differences. In the discussion, we identify the key emerging elements and organise them in an analytical framework (Fig. 1), which helps to highlight the key nodes where adaptations happen, the contextual drivers of adaptation, and the organisational elements that enable or facilitate adaptation and may sustain PBF programmes, focusing on ‘hardware’ and ‘software’ elements of the health system [50].

The inner circle in Fig. 1 presents the PBF ‘principles’ (i.e., autonomy for health facilities, payment according to verified performance, contractual relationships, separation of functions, community engagement, equity bonuses), as they have become codified over time [2, 51, 52]. These PBF ‘principles’ have helped to identify functions and roles, and also to promote PBF as a coherent approach, building on experience as implementation progressed across countries. However, the rigid codification of PBF carries a risk in terms of fossilisation of the approach which becomes less adaptable to context.

The second concentric circle in Fig. 1 highlights that, despite the ‘principles’, PBF can be, and has been, adapted in a pragmatic way to respond to the specificities of local contexts (the ‘contextual drivers of adaptation’ are represented in the third circle in Fig. 1). The challenging environments that we analysed did require a high degree of adaptation and innovation, and we found a number of examples in our analysis (in Fig. 1, examples are mapped against the principles they contravene by using the same colour). Our cases show that: functions have been combined where reliable institutions to carry them out were lacking; contracting of the local regulator by implementing agencies was introduced as a measure to cope with the absent central state; changes have been made to verification procedures including foregoing verification when it was risky to carry it out; non-performance based, and in some cases input-based, support was provided under the PBF programme (rather than in parallel by other programmes) for the rehabilitation and construction of destroyed infrastructure; and direct intervention of the implementers for the procurement of drugs and supplies (sometimes in collaboration with other NGOs) or for hiring staff occurred in the absence of functioning markets. Adaptations have also been made to respond to crisis by providing free care to IDPs, even in contexts where free care is not an official government policy. Where communities had been disrupted by violence and displacement, their engagement and contracting for verification did not work as envisaged (similarly to other contexts [53]), but communities were contracted in other pragmatic ways to support health service delivery, for example by providing labour and materials for reconstruction and rehabilitation of facilities. Some of these adaptations have also been observed in other countries. For example, in Sierra Leone during the Ebola epidemic payments were done without verification [54], and increasing PBF bonus to subsidise free care for IDPs in South Kivu represented an early example of what is now a practice in other PBF projects, including for example in CAR and Cameroon [51, 55, 56]. However, the literature so far rather reports these adaptations but has not analysed them in relation to the humanitarian and crisis context.

It is also relevant to explore what enabled and facilitated these adaptations (highlighted in the outer ring of Fig. 1). Our analysis shows that the decision space and the margin of manoeuvre to adapt available to implementers is affected by their funder(s)’ requirements and funding levels, but also by their organisational capacities, technical knowledge, interface with communities, social accountability, individual influences, and importantly national leadership. We find that, in difficult environments, the risk related to PBF implementation is pushed on to the implementing organisations. As a result, they can only survive if they draw on their resources, which include financial, but also technical and relational (local organisation, trust and knowledge). In particular, organisational flexibility, in terms of budget levels, budget use but also mindsets, management structures and innovation capacity plays a key role in allowing adaptations and changes to PBF in order to cope with the challenges. Similarly, long-term relationships within the area and local staff with good contextual knowledge and links also proved to be essential. In our case studies in South Kivu and CAR, Cordaid appears to have built considerable social capital, which it was able to draw on for protection and continued functioning, especially when the PBF programme’s rules and regulations allowed for more flexibility in adaptation. In northern Nigeria, the recognised leadership of the local government agency in charge of PBF implementation was essential to ensure a coordinated and adapted response to the crisis. PBF also provided a ‘structure’ based on the distribution of roles and functions that PBF entailed, that helped the SPHCDA to organise, coordinate and support the provision of healthcare services, during the crisis and especially in the IDP camps.

In contrast with the Nigerian context, in the environments of CAR and South Kivu, governance is marked by multiple actors, power imbalances, fragmentation, and competing agendas [18]. The lack of well-defined and effectively enforced policies creates a de facto policy (and financing) void at local level, which allows PBF to be adapted and implemented more freely. In these contexts, PBF is not necessarily clashing with the (absent) policies and strategies, for example, concerning facility autonomy and use of funds, in contrast to what noted in other countries, such as Cameroon [57]. However, given the fragmentation of funding flows and the competition for funding, clash and negotiations occur between PBF implementers and other external players. Additionally, in the political void, PBF implementers with resources can become ‘policy-makers’ themselves, by contracting the local authorities and contributing to (re)define their roles and tasks, as in South Kivu. This approach may be effective to ensure funding and (some) accountability, and therefore improve service delivery, but its longer term legacy on state-building is less well documented. We recognise, however, that this is an alternative to other approaches to dealing with the challenges related to the weakness and underfunding of the local state, which are also sub-optimal. Other options include bypassing the higher (provincial) authorities to deal directly with the Zones or even the facilities, as done by most humanitarian actors [58]. This creates tensions at facility level and further weakens the provincial authorities. Another option is to select one or few individual champion(s) with extensive patronage networks or political weight and motivate them (financially or otherwise) to push the donors’ or NGOs’ agenda - again bypassing formal institutions and reinforcing the patronage system [25].

Additionally, in the case of South Kivu and CAR, alongside the “privatisation from within” [26], the weakness of local institutions and lack of ‘veto points’ from public authorities may have also contributed to making the introduction of PBF concepts relatively easy. Indeed, we find that, in those cases, processes of PBF adoption were externally driven, as highlighted in the literature in particular with reference to fragile states [5, 59, 60]. Nigeria presents a different context as the federal authorities and structures remained in place and only parts of the country were conflict-affected. As a consequence, the introduction of PBF in Nigeria was more formally directed, and the MoH leadership was strong enough to demand national ownership.

Our study has certain limitations. In terms of data collection, because participants’ identification was based on initial contacts provided by implementing agencies, some degree of respondent bias is possible. Generally, the sample is unbalanced towards those involved in PBF implementation rather than their counterparts, although we did try to capture the views of other organisations operating in the area and of the MoH at different levels. Additionally, our focus was essentially on central/federal and state/provincial levels, as well as district/zonal where possible, but we did not capture the views of the service providers or of the communities they served. Finally, because of lack of specific data on the effects of PBF on health outcomes and health system elements, we were not able to complete the last element of the study which was guiding us, relating to effects [13]. Overall, this study remains exploratory in nature.

## Conclusions

This study explores the introduction and implementation of PBF in three conflict-affected settings: South Kivu in the DRC, the CAR, and Adamawa State in northern Nigeria. It looks at how and why PBF is adopted in such settings, how it is adapted to them, what drives and what facilitates these adaptations. The case studies and their comparison provide relevant insights on a largely unexplored topic, which is of high importance for improving both our theoretical understanding of PBF and its operational practice.

In particular, our study adds to the literature on PBF implementation, with specific attention to the influence of context and contextual adaptations, which has been very rarely discussed in the literature so far. The findings point to the importance of pragmatic adaptation in PBF design and implementation that is necessary to reflect the specificities of each context. While conflict-affected settings represent an extreme case of challenging environments (which perhaps explains why adaptations are made, while PBF programmes in other settings appear more rigid), further research could confirm that our conclusions are applicable beyond these contexts. In particular, the elements that we identified as facilitating or enabling adaptations (such as organisational flexibility, local staff and knowledge, and embedded long-term partners) appear to be relevant across settings. They are useful to spark a reflection among PBF donors and implementers on the relevance of incorporating, reinforcing and building on those elements when designing and implementing PBF programmes. Additionally, although the elements identified in our cases are likely to be context-specific, the structure of the framework that we develop could represent a useful tool for further analytical work in different contexts.

## Additional file

Additional file 1: Topic guide. (DOCX 46 kb)

## Footnotes

1 It is interesting to note that, even if Cordaid’s health PBF programme in South Kivu has been discontinued, the AAP retains its purchasing role for PBF in other sectors, and also for the new World Bank-funded PBF programme in the health sector.
