# Supplementary material for: Performance-based financing in three humanitarian settings: principles and pragmatism
Source: Confl Health. 2018 Jun 27;12:28. doi: 10.1186/s13031-018-0166-9 (PMC6020366; doi:10.1186/s13031-018-0166-9)
Supplement: Supplementary file 1 — Topic guide. (DOCX 46 kb) [file 13031_2018_166_MOESM1_ESM.docx]

**Topic guide**

A. Note details of participants before interview:

| 1. Interviewee ID |  |
| --- | --- |
| 1. Date of Interview |  |
| 1. Gender | Male □ Female □ |
| 1. Title of interviewee |  |
| 1. Institution / Organization / Department |  |
| 1. Central, or Region & District |  |

Ask some general questions about how long the person has been in post, the time period of his/her experience with PBF, the context(s) of which he/she has experience, etc., to help with adapting the questions.

B. Questions *(tailor according to background of KI and the PBF programme(s )of which they have knowledge and experience – could be one or more than one)*

- Background on fragility
  - Can you briefly describe the key events with relation to conflict, violence and/or fragility in your setting? (i.e., history of fragility and conflict)
  - In your view, what are the main features of fragility? (*probe*: very broad description of the context in relation to fragility, could include descriptive elements of: acute or chronic violence, ‘statelessness’ (lack of central government oversight and stewardship), consequences of conflict on health system (lack of infrastructure and equipment, health workforce crisis, …), recovery needs, etc.)
  - What are – in your opinion – the most important issues/constraints and perhaps opportunities defined by “fragility” that influence healthcare service delivery?
- Background on PBF
  - Where is PBF implemented?
  - What are the key design features? i.e., in terms of geographical coverage, population coverage (age and income groups), providers’ coverage (primary level, secondary/tertiary level, district management teams, community health workers, etc. as well as public, private, non-for-profit), service coverage (indicators included); verification procedures; separation of functions; management autonomy of facilities; etc.
  - What are the key health financing mechanisms (channelling, payer, decision making on payment, autonomy in payment/ utilisation of PBF-funds, …); human resources arrangements (payment of HF team or individuals, distribution RBF funds between staff, attraction new staff), governance arrangements (new Contracting & Verification Agency; relation demand-supply, community involvement, split between provider/ purchaser/ regulator/ verificator, …)
  - Since when has PBF been implemented? Pre-pilot – to design the model? Who introduced the idea?
  - What is your view on who were the main actors involved in the introduction of PBF? How were they positioned (in favour, against, neutral in relation to PBF)? What was their relative power and influence and how was it exercised?
  - Why was PBF considered a potentially useful option and/or why was it opposed?
  - Do you think that PBF was introduced as part of broader health system / health financing reforms and discussions? If so, what was such context (e.g. introduction of free health care policies, decentralisation policies, etc.)?
- How has PBF been adapted to respond to humanitarian crisis and/or early recovery efforts? *(ask for each PBF programme that the KI is involved in / has knowledge of)*
  - Was PBF modified already during the design phase or rather at implementation stage?
  - What was the process of modification/ was it based on evidence (literature, other experts, ….) or on power relations?
  - Which key elements of PBF have been modified? and why (i.e., which specific challenges and problems did the adaptations aim to address)?
  - Which individuals and organizations were involved in the adaptation of PBF? (including your role and that of your organization)
  - What was the relative position of these organizations in terms of support / opposition to PBF changes? Who were the most relevant actors to drive the changes?
  - Which elements contributed to outline the options available and make a decision on the adaptations to introduce? (for example, evidence, previous experience in other settings, needs assessment, urgency to address an issue, etc.).
- What are the main advantages and what are the challenges of implementing the adapted PBF design?
  - What are your views about whether and how the adaptation successfully addressed the challenges originally identified?
  - Where there any unexpected positive/negative effects of PBF under the new design?
  - Are there any outstanding challenges which the adaptation did not address? Why?
  - Why has PBF worked (or not) as planned in your setting? *(please, provide any reports of assessments and evaluations which may have been carried out)*
  - How are/were the core mechanisms of PBF modified or influenced by the context in disrupted settings? (e.g., was verification possible?, did facility have enough autonomy? Where they able to effectively utilise the funds available to improve coverage and quality of service?, etc.)
  - In your opinion, which underlying conditions and/or features of the context may have supported or hindered the implementation and the effectiveness of PBF?

🡪 such conditions/features may be related to the health sector, its policies and practices – including, for example, the interaction between PBF and free health care, the integration of PBF rules into management of facilities and health workforce, structural barriers to improving coverage and quality of services that which PBF could not address etc.);

🡪 Or could relate to the broader context (e.g., elements of fragility and conflict, lack of trust among key actors, lack of stewardship and accountability (“structure”) driven by national level, role of/reliance on external actors at local level (NGOs), etc.).

- - What are the effects of PBF and PBF’s adaptations on the broader health system (see elements below) and on health system strengthening:
    - governance/ institutional frameworks,
    - partnerships working at operational level,
    - HMIS,
    - public-private service provision,
    - purchaser-provider arrangements,
    - supply and demand for healthcare
